# Supplementary material for: Symmetry-constrained generation of diverse low-bandgap molecules with Monte Carlo tree search
Source: Chem Sci. 2025 May 10;16(23):10503–11. doi: 10.1039/d4sc08675a (PMC12068513; doi:10.1039/d4sc08675a)
Supplement: SC-016-D4SC08675A-s001 [file SC-016-D4SC08675A-s001.pdf]

Supplementary Information (SI) for Chemical Science.
This journal is © The Royal Society of Chemistry 2025

Journal Name

ARTICLE TYPE

Cite this: DOI: 00.0000/xxxxxxxxxx

Supplementary Information

Symmetry-Constrained Generation of Diverse Low-Bandgap Molecules with Monte Carlo Tree Search

Akshay Subramanian,[a] James Damewood,[a] Juno Nam,[a] Kevin P. Greenman,[b] Avni P. Singhal,[a] and Rafael Gómez-Bombarelli [a‡]

1 Acquisition Function for AL

Expected improvement is a bayesian acquisition function that builds upon probability of improvement. We reproduce a brief excerpt of the derivation below, but we refer readers to Kamperis[1] for a more detailed discussion.

1.1 Probability of Improvement (PI)

Given a function f, our task is to estimate the probability that $f(x) > f(x^{\star})$ where $x^{\star}$ is a previous optimum. If improvement is defined as,

$$I(x) = max(0, f(x) - f(x^{\star})),$$

where $f(x)$ is treated as a random variable following the gaussian distribution $\mathcal{N}(\mu, \sigma^2)$, then using the reparameterization trick, we can rewrite $I(x)$ as

$$I(x) = max(0, \mu(x) + \sigma(x)z - f(x^{\star})), z \sim \mathcal{N}(0,1)$$

Then the probability of improvement

$$PI(x) = Pr(I(x) > 0) = \Phi(\frac{\mu(x) - f(x^{\star})}{\sigma(x)}),$$

where $\Phi(z) = CDF(z)$.

1.2 Expected Improvement (EI)

EI is the expected value of improvement $I(x)$, which unlike PI, gives us an estimate of the magnitude of improvement rather than just the probability of improvement.

It is defined as

$$EI(x) = \int_{-\infty}^{\infty} I(x)\varphi(z)dz = \int_{-\infty}^{\infty} max(0, f(x) - f(x^{\star}))\varphi(z)dz,$$

where $\varphi(z) = \frac{1}{\sqrt{2\pi}} exp(-z^2/2)$. The integral can be split as

$$EI(x) = \int_{-\infty}^{z_0} I(x)\varphi(z)dz + \int_{z_0}^{\infty} I(x)\varphi(z)dz$$

The first term is zero since $I(x) = 0$.

$$\begin{aligned} EI(x) &= \int_{z_0}^{\infty} (\mu + \sigma z - f(x^{\star}))\varphi(z)dz \\ &= \int_{z_0}^{\infty} (\mu - f(x^{\star}))\,\varphi(z)\,\mathrm{d}z + \int_{z_0}^{\infty} \sigma z \frac{1}{\sqrt{2\pi}} e^{-z^2/2}\,\mathrm{d}z \\ &= (\mu - f(x^{\star})) + \sigma\varphi(z_0) \\ &= (\mu - f(x^{\star}))\,\Phi\left(\frac{\mu - f(x^{\star})}{\sigma}\right) + \sigma\varphi\left(\frac{\mu - f(x^{\star})}{\sigma}\right), \end{aligned}$$

where Φ and φ are the cumulative distribution function (CDF) and probability density function (PDF) of a normal distribution, respectively.

2 Synthetic Complexity and Bandgap

The ideal behavior of a generative model on our task is to minimize synthetic complexity and bandgap simultaneously. We investigated the relationship between these two quantities, and performed comparisons against another fragment-based generative model, junction tree variational autoencoder (JT-VAE)[2] which also uses fragment vocabulary from the same patent-mined dataset. In Figure 2(a), we see that random samples from MCTS are already left-shifted in bandgaps in comparison to random samples from a trained JT-VAE model. This suggests that the chemical space explored by MCTS is already more targeted towards the property of interest even before any optimization is performed. Since both models obtain fragment vocabulary from the same patent dataset, this indicates a positive influence of our defined grammar (MDP described in main text) towards minimizing bandgaps.

We measure synthetic complexity of molecules with synthetic complexity score (SCScore).[3] In (b) and (c), we show that SCScore exhibits a positive trend wrt molecular mass, and a negative trend wrt bandgap, respectively. We can also see from these plots that JT-VAE generates a significant amount of candidates with low molecular mass, that also have low SCScore, but are sub-optimal in property space (have high bandgaps). Therefore, we are interested in the pareto-front of SCScore and bandgap rather than just minimizing either one. We see from (d) that the trend line between SCScore and bandgap for JT-VAE has a steeper slope in comparison to MCTS, indicating that a decrease in bandgap has

[a] *Department of Materials Science and Engineering, Massachusetts Institute of Technology, Cambridge, MA, USA.*
[b] *Department of Chemical Engineering, Massachussets Institute of Technology, Cambridge, MA, USA.*
‡ Correspondence to: Rafael Gómez-Bombarelli <rafagb@mit.edu>.

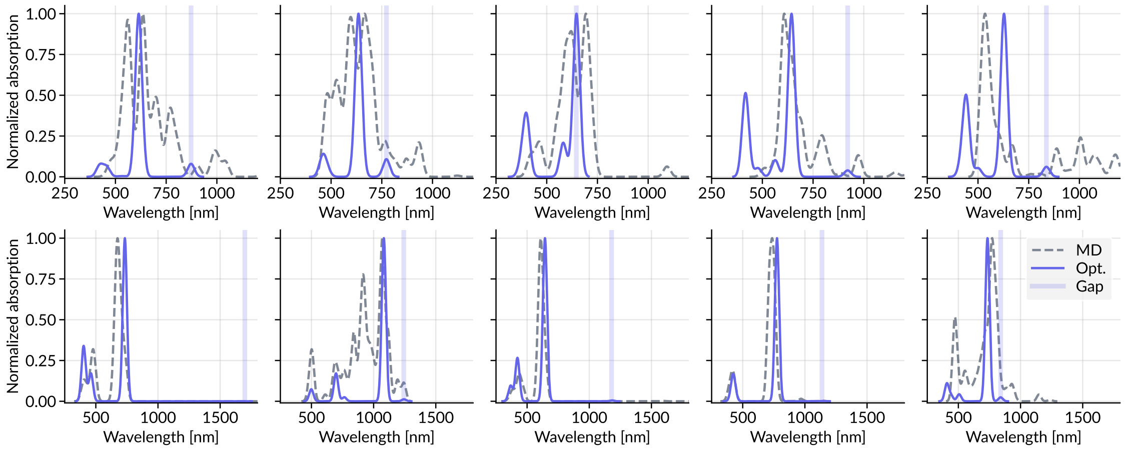

Fig. 1 Computed absorption spectra for final candidate molecules. The spectra for molecules are ordered as shown in Figure 4 in the main text, with Y6 derivatives in the upper row and patent-extracted fragment designs in the lower row. Solid lines represent single-point TD-DFT calculations at the optimized geometry, while dotted lines represent statistically averaged TD-DFT spectra from molecular clusters sampled from MD simulations. The translucent vertical lines indicate the band gaps from the single-point calculations. The spectra are normalized so that the maximum absorption corresponds to 1.0.

a sharper increase in SCScore. This suggests that MCTS has the capability of generating lower bandgap candidates while not compromising on SCScore, in comparison to JT-VAE.

3 Finite Temperature and Aggregation Effects with Coupled MD/TD-DFT Calculations

3.1 Computational Methods

We combined MD simulations with TD-DFT calculations to approximate the effects of finite temperature and molecular aggregation on absorption spectra. Following Kupgan *et al.* [4], we simulated an amorphous morphology for the molecules. Starting with the optimized geometries presented in the main text, we used PACKMOL[5] to pack 200 structures in a cubic box at a low density (~ 0.1 g/cm^3). The OPLS-AA force field[6] was used via the LigParGen server[7]. The system was equilibrated for 30 ns at 650 K, cooled to room temperature (300 K) at 10 K/ns, and subjected to a 30 ns production run at 300 K. All MD simulations were performed under the NPT ensemble at 1 atm, using GROMACS 2023[8]. From the last 20 ns of the production run, we randomly selected a molecule and included all neighboring molecules within a 5 Å radius. CHELPG charges[9] were computed for each neighboring molecule, and TD-DFT calculations were performed for the selected molecule with the neighboring point charges. For electronic structure calculations, we used the same settings as in the main text. The absorption spectra were averaged over 10 clusters to obtain a statistical estimate.

Two of the patent-derived molecules (the first and fifth molecules in Figure 4 of the main text) contain an isocyanide moiety, which could not be parametrized correctly using the LigParGen server. Therefore, for these two molecules, we substituted the isocyanide (–NC) functional group with cyanide (–CN) for this analysis. This substitution does not impact the band gap calculations significantly, with differences in bandgaps for the optimized geometries of only 2 and 6 meV, respectively, indicating negligible effects on the result analysis.

3.2 Absorption Spectra of Final Candidates

The absorption spectra for the designed molecules are presented in Figure 1, with the Y6 derivatives in the upper row and patent derivatives in the lower row. The vertical lines indicate the TD-DFT band gap for the optimized geometries, as reported in Figure 4 of the main text. The MD/TD-DFT pipeline results for the primary absorption peak locations are consistent with those from static (optimized) TD-DFT calculations, though the MD/TD-DFT spectra exhibit broadening, likely due to thermally accessible conformers and neighboring molecules. Note that the reddest absorption peak from TD-DFT corresponding to the band gap might not accurately reflect the absorption spectra when its oscillator strength is very low. This limitation, inherent to the design objective focused solely on the band gap, indicates the need to consider transition probability in future designs. Nonetheless, the band gap serves as a robust design objective, and this result demonstrates that our MCTS design pipeline effectively produces low-bandgap molecules.

4 Preprocessing of Patent-Mined Molecules

Before fragment decomposition was performed, we performed some minimal preprocessing steps to ensure that the molecules extracted from patents are representative of the chemical space we want to explore. We first performed an RDKit filter, to ensure that only SMILES strings representing valid molecules are retained, followed by an element filter, which retained only fragments containing the following elements: `C`, `O`, `N`, `H`, `Cl`, `Br`, `S`, `F`, `I`, and `Si`. We finally removed molecules that do not contain any aromatic atoms.

5 Correlation Between DFT and Experimental Bandgaps

We performed TD-DFT calculations on a subset of experimental molecules from the list shown in Figure 3 for which we had access to experimentally measured bandgaps. The correlation plot

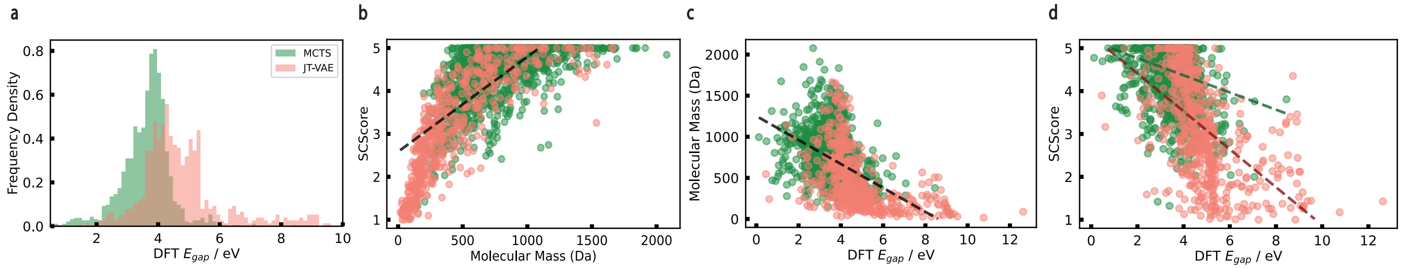

Fig. 2 Synthetic complexity score (SCScore) and comparison against JT-VAE. (a) Bandgap distributions of randomly sampled candidates from MCTS and JT-VAE. (b), (c), and (d) show trends of SCScore vs Molecular Mass, Molecular Mass vs Bandgap, and SCScore vs Bandgap, respectively. All trend lines were obtained through least-squares fit.

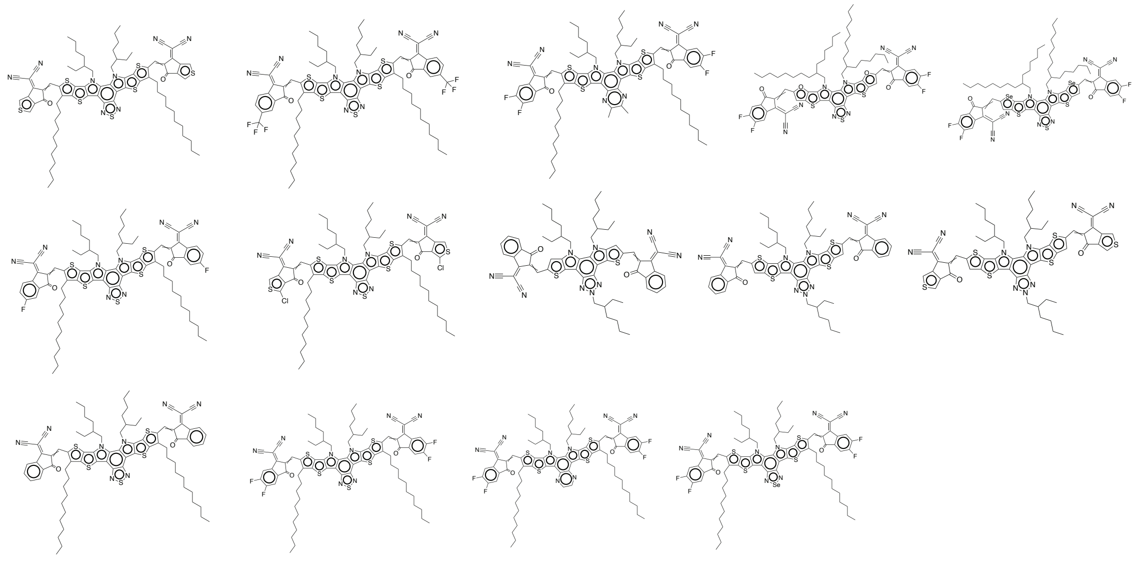

Fig. 3 Some popular experimentally used acceptor molecules. These were chosen from Lu *et al.*[10].

between experimental and DFT values are shown in Figure 4. We see R^2 value of 0.57, and a Spearman rank correlation coefficient (SRCC) of 0.74. While the magnitudes are not well calibrated, the trends of experiments are well-captured by TD-DFT, making minimization a suitable objective for MCTS as opposed to optimization for targeted bandgap values. As a future extension of this work, it could be possible to fit a calibration function between TD-DFT and experimental bandgaps and use the calibrated rewards for training. While it could be somewhat reasonable to extrapolate such calibrations for the Y6 MDP, it might be more challenging to obtain representative experimental examples for the patents MDP on which a calibration fit can be performed.

The mismatch in magnitudes between TD-DFT and experiments can be caused by several factors including (but not limited to) 1) finite temperature effects, 2) molecular aggregation effects from experimental values being measured on thin films, 3) inherent error in TD-DFT functional.

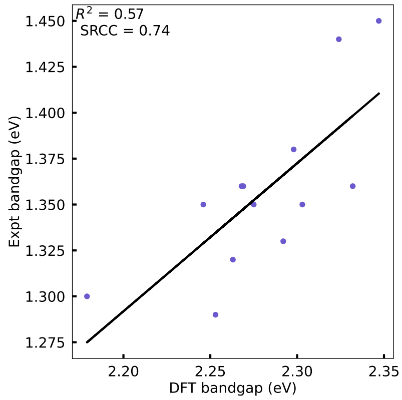

Fig. 4 Correlation between TD-DFT and experimental bandgaps. R^2 score and Spearman rank correlation coefficient are shown on the top left.

6 Fragment Attribution

One of the key advantages of having a fragment-based approach such as MCTS is the potential to draw some correlations between the presence/absence of fragments and property scores. Figure 5 shows the fragments that were present in molecules during MCTS training, the average chemprop-predicted bandgap arising from those fragments, and the frequency of their observation during training. We can see in the Y6 MDP that typically the polyene pi-bridges have a lower average bandgap in comparison to aromatic ring-based bridges such as thiophene, benzothiadiazole, and pyrrole. This can potentially arise from the increased planarity

and therefore a maintenance of stronger orbital overlap and pi-conjugation in the former in comparison to the slightly more non-planar ring conformations and steric effects in the latter. This allows for stronger intra-molecular charge transfer effects between the electron-donating cores and the electron-withdrawing end-groups. Among heteroatom modification choices (`pos0`, `pos1`, `pos2`, `pos3`), it is evident that expanding the ring size with carbon and nitrogen atoms is not as favorable as simply modifying the hetero-atom in the five-membered ring, which can be expected given that five-membered rings pack larger charge densities and therefore promote more favorable intra-molecular interactions, and also reduce steric effects and increase planarity. Similar increasing effects on bandgap have been observed due to out-of-plane twisting in Bijleveld *et al.* [11] when 6-member rings were used alongside 5-member rings in polymers.

In the patent MDP, it is very apparent that fused-ring cores that maintain stronger electron density and conjugation are much more favorable in comparison to non-conjugated/partially-conjugated cores. [12,13] Since the chemical space is much larger in the patent MDP, we can see that our limited computational budget for training prevented us from exploring pi-bridges and end-groups to frequencies that could make attribution statistically significant. While this is outside the scope of this paper, more large scale training that leads to a thorough exploration of the chemical space can be useful for further insights and analysis.

The above analysis with 1-grams of fragments captures property impacts of individual fragments, but not the inter-fragment correlations. To model these dependencies, we also analyzed the lowest bandgap 2-grams (pairs) and 3-grams (triplets). We show results for the core/end-group pair and core/pi-bridge/end-group triplet in Figure 6. It can be seen that the lowest bandgaps are achieved with pairs of electron donating fused-ring cores and electron withdrawing end-groups, confirming that push-pull effect is beneficial towards low bandgaps. Similarly, among triplets, the best cases are where conjugation is maintained between cores, pi-bridges, and end-groups. The highest bandgaps are achieved when the conjugation pathway is broken in some way.

7 Structural Modifications

For the fifth molecule shown in Figure 4 of the main text, we identified that errors in atomic connectivity were introduced during the xTB optimization step. We fixed the geometry by bypassing the xTB optimization step altogether. We instead directly performed BP86 DFT optimization on the five lowest energy conformers obtained from RDKit ETKDG algorithm, and performed the TD-DFT calculation on the lowest energy BP86 conformer.

As mentioned in Section 3 of the main text, we performed TD-DFT calculations on methyl-substituted versions of reactive molecules to confirm that the bandgap does not change significantly after substitution. Bandgaps of second, fourth, and fifth molecules changed very minimally from 0.998, 1.09, and 1.470, to 0.978, 1.049, and 1.470 eV respectively.

8 Reactive Positions in Practice

While we explain the algorithm in Section 2.1.1 of the main text using IDs belonging to $[0, N)$, in the implementation, we use Helium isotopes with IDs ranging from $[100, 100+N)$ where N is the number of fragments in the vocabulary, to preserve reactive position identities in the RDKit `Mol` object. The range was chosen arbitrarily but to be large enough so that there are no clashes in atomic mass with other elements in the dataset.

9 Fragments in Y6 MDP

We illustrate the fragments along with their reactive positions in Figure 7.

Notes and references

1 S. Kamperis, *Lets Talk Sci. https://ekamperi. github. io/machine% 20learning/2021/06/11/acquisition-functions. html*, 2021.
2 W. Jin, R. Barzilay and T. Jaakkola, International conference on machine learning, 2018, pp. 2323–2332.
3 C. W. Coley, L. Rogers, W. H. Green and K. F. Jensen, *Journal of chemical information and modeling*, 2018, **58**, 252–261.
4 G. Kupgan, X. Chen and J.-L. Bredas, *Materials Today Advances*, 2021, **11**, 100154.
5 L. Martínez, R. Andrade, E. G. Birgin and J. M. Martínez, *Journal of Computational Chemistry*, 2009, **30**, 2157–2164.
6 M. J. Robertson, J. Tirado-Rives and W. L. Jorgensen, *Journal of chemical theory and computation*, 2015, **11**, 3499–3509.
7 L. S. Dodda, I. Cabeza de Vaca, J. Tirado-Rives and W. L. Jorgensen, *Nucleic acids research*, 2017, **45**, W331–W336.
8 M. J. Abraham, T. Murtola, R. Schulz, S. Páll, J. C. Smith, B. Hess and E. Lindahl, *SoftwareX*, 2015, **1**, 19–25.
9 C. M. Breneman and K. B. Wiberg, *Journal of Computational Chemistry*, 1990, **11**, 361–373.
10 B. Lu, J. Wang, Z. Zhang, J. Wang, X. Yuan, Y. Ding, Y. Wang and Y. Yao, *Nano Select*, 2021, **2**, 2029–2039.
11 J. C. Bijleveld, B. P. Karsten, S. G. Mathijssen, M. M. Wienk, D. M. de Leeuw and R. A. Janssen, *Journal of Materials Chemistry*, 2011, **21**, 1600–1606.
12 W. Liu, S. Xu, H. Lai, W. Liu, F. He and X. Zhu, *CCS Chemistry*, 2023, **5**, 654–668.
13 B. Schweda, M. Reinfelds, P. Hofstadler, G. Trimmel and T. Rath, *ACS applied energy materials*, 2021, **4**, 11899–11981.

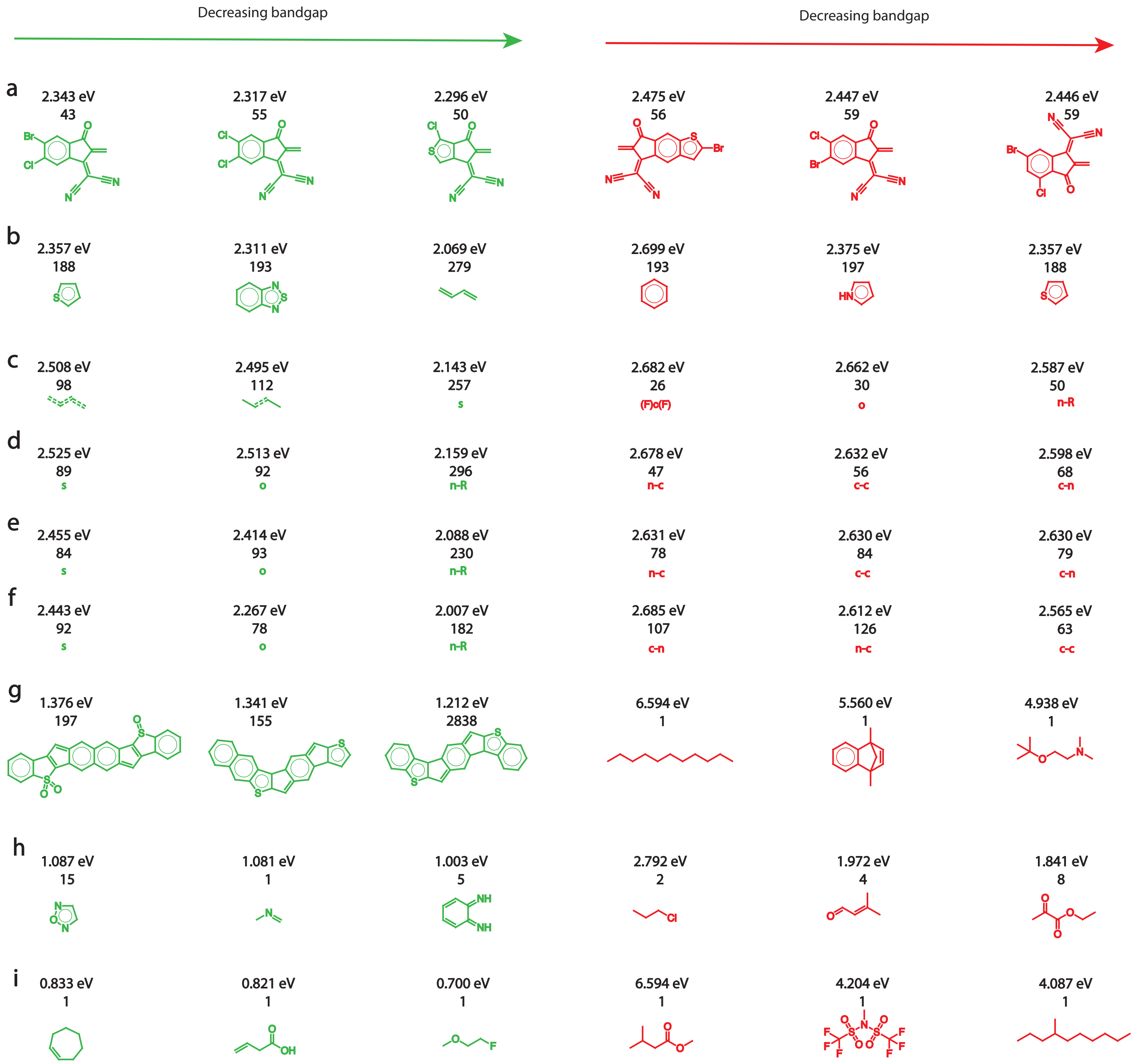

Fig. 5 Fragment attribution 1-gram. (a-f) represent end-groups, pi-bridges, pos0, pos1, pos2, and pos3 actions respectively of the Y6 MDP, while (g-i) represent cores, pi-bridges, and end-groups respectively of the patent MDP. The numbers shown above each fragment are the average chemprop-predicted bandgap of all molecules formed using that fragment during training, and the frequency of observation of that fragment. Fragments in green and red represent the best and worst 3 fragments respectively in average bandgaps.

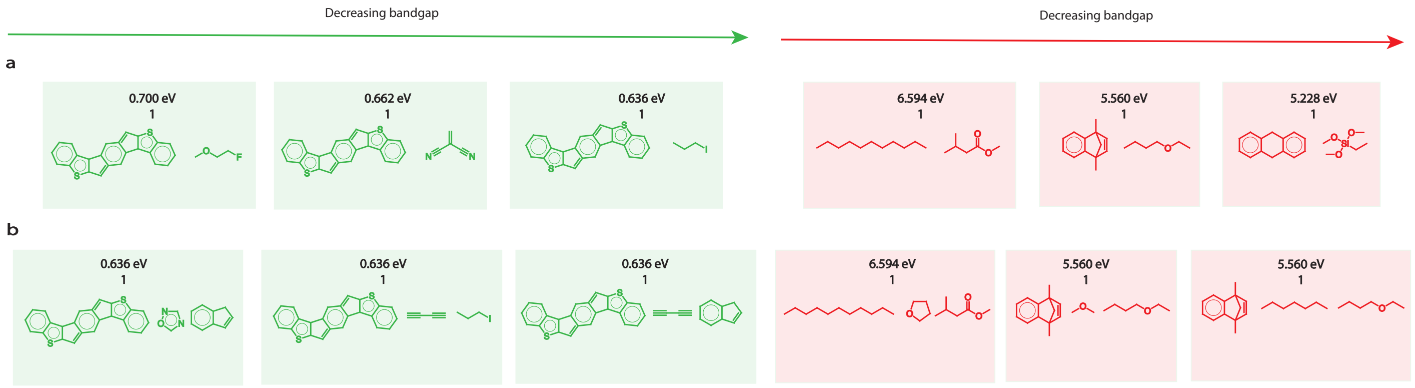

Fig. 6 Fragment attribution 2-grams and 3-grams. (a) represents core and end-group pairs, and (b) represents core, pi-bridge, and end-group triplets. Color scheme and numbers shown in the figure follow the same conventions as Figure 5.

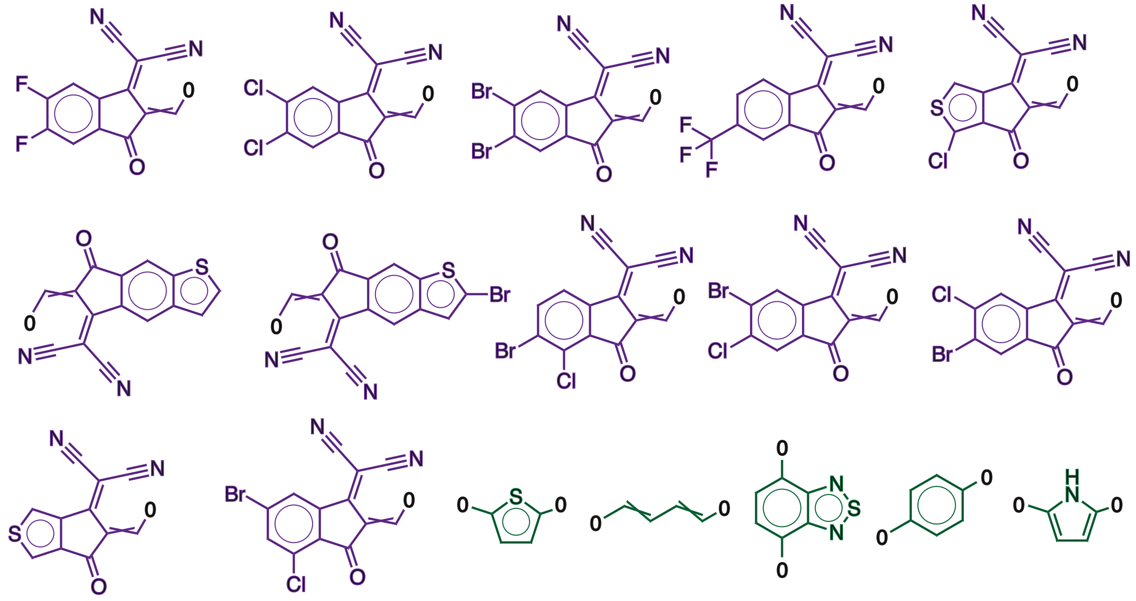

Fig. 7 Fragments used in Y6 MDP end-groups (purple) and pi-bridges (green). Core-modification actions are implemented as string replacements, the list of which can be found in the GitHub repository. We also omit illustrating alkyl chain actions here since methyl group is the only choice explored.
